# Managing Stroke Prevention in Hypertrophic Obstructive Cardiomyopathy (HOCM) Patients Without Confirmed Persistent Atrial Fibrillation: A Dilemma of Anticoagulation

**DOI:** 10.7759/cureus.46612

**Published:** 2023-10-07

**Authors:** Christine Henen, Elise A Johnson, Sergio Sokol

**Affiliations:** 1 Internal Medicine, St. John's Episcopal Hospital, Far Rockaway, USA; 2 Internal Medicine, Ross University School of Medicine, Miramar, USA; 3 Cardiology, St. John's Episcopal Hospital, Far Rockaway, USA

**Keywords:** persistent atrial fibrillation, paroxysmal afib, stroke, atrial fibrillation (af), hypertrophic obstructive cardiomyopathy (hocm)

## Abstract

Hypertrophic obstructive cardiomyopathy (HOCM) is a genetic cardiovascular disorder characterized by the thickening of the heart muscle, particularly the left ventricle. It is a leading cause of sudden cardiac death in young individuals. HOCM is associated with various complications, including arrhythmias and an increased risk of stroke.

Patients with HOCM are at an increased risk of stroke due to the development of atrial fibrillation (AFib), a common arrhythmia observed in HOCM. AFib can result in the formation of blood clots in the atria, which may subsequently embolize the brain, causing a stroke. However, not all HOCM patients develop persistent AFib, leading to uncertainty regarding the appropriate management of stroke prevention in these cases.

This case study aims to explore the management of recurrent cerebrovascular events (CVA) in a patient with HOCM who does not have confirmed persistent AFib. The argument revolves around whether anticoagulation should be offered for secondary stroke prevention in HOCM patients without a confirmed diagnosis of persistent AFib.

## Introduction

We present a case study with a challenging scenario regarding managing stroke prevention in a patient with hypertrophic obstructive cardiomyopathy (HOCM) who does not have a confirmed diagnosis of persistent atrial fibrillation (AFib). The decision to initiate anticoagulation in such cases requires careful consideration, weighing the potential benefits against the risks associated with anticoagulant therapy. To address this dilemma, it is crucial to review the existing literature on stroke risk in HOCM patients, particularly those without confirmed persistent AFib, as well as the evidence supporting or questioning the use of anticoagulation in this specific patient population.

Studies investigating the risk of stroke in HOCM patients have consistently shown an increased risk of stroke in those with associated atrial fibrillation [1]. However, limited research focuses explicitly on the stroke risk in HOCM patients without confirmed persistent AFib. Despite the lack of specific studies addressing this population, research on anticoagulation for stroke prevention in patients with cardiomyopathies, including HOCM, without confirmed persistent AFib suggests potential benefits [2]. These studies generally indicate that anticoagulation may be beneficial in reducing stroke risk in this patient population.

Advocates for anticoagulation in HOCM patients without confirmed persistent AFib argue several points. First, HOCM patients, even without confirmed persistent AFib, are still at an elevated risk of stroke due to underlying cardiac abnormalities and potential paroxysmal arrhythmias. Second, the pathophysiology of stroke in HOCM patients without confirmed persistent AFib is similar to those with persistent AFib, with the formation of thrombi in the atria occurring during paroxysmal arrhythmias, leading to embolic strokes. Third, arrhythmias, including AFib, may be underdiagnosed in HOCM patients without persistent AFib. Using loop recorders or prolonged monitoring may detect intermittent arrhythmias that could contribute to stroke risk [2]. Finally, anticoagulation has proven efficacy in reducing stroke risk in patients with persistent AFib, and extending its use to HOCM patients without persistent AFib could offer similar benefits.

Counterarguments against anticoagulation in HOCM patients without confirmed persistent AFib include the lack of specific studies addressing this patient population, making it challenging to establish clear recommendations [3]. Additionally, anticoagulation carries a risk of bleeding [1], which may be a concern in patients without confirmed persistent AFib who have a lower stroke risk than those with persistent AFib. Some argue for an individualized approach, considering the patient's overall risk profile, including other risk factors for stroke, to determine the need for anticoagulation [1].

## Case presentation

Patient presentation and admission details

The patient is a 64-year-old retired Indian female with a significant medical history of hyperlipidemia, hypertension, remote history of gastrointestinal bleed, and a diagnosis of hypertrophic obstructive cardiomyopathy (HOCM) [3]. She presented to the emergency department with new-onset left upper extremity weakness and numbness, which resolved spontaneously. Subsequently, she experienced difficulties speaking and chest pain, but these symptoms also resolved. The following day, the patient woke up with left facial droop and left upper extremity weakness.

Medical history and relevant findings

**Figure 1:**
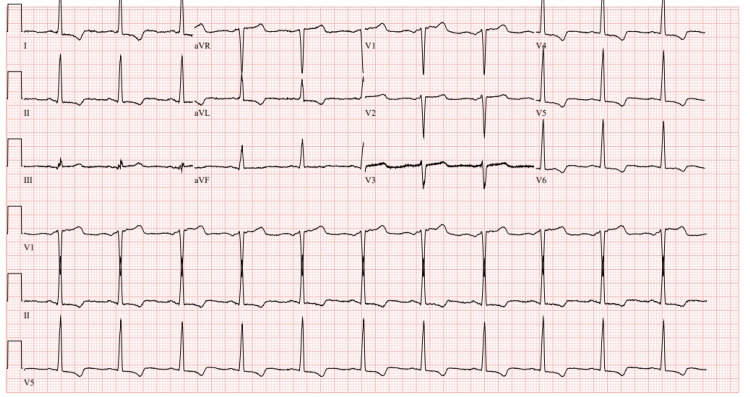
EKG on admission NSR, LVH with lateral T wave inversion, junctional rhythm and LVH with repolarization abnormalities.

The patient's medical history includes hyperlipidemia, hypertension, remote history of GI bleed, and a diagnosis of HOCM [3]. Additionally, a family history of both HOCM and stroke. Laboratory tests conducted on the patient did not reveal any significant abnormalities. The patient's electrocardiogram (EKG) at that time demonstrated normal sinus rhythm (NSR), left ventricular hypertrophy (LVH) with lateral T wave inversion, junctional rhythm, and LVH with repolarization abnormalities (Figure 1).

**Figure 2:**
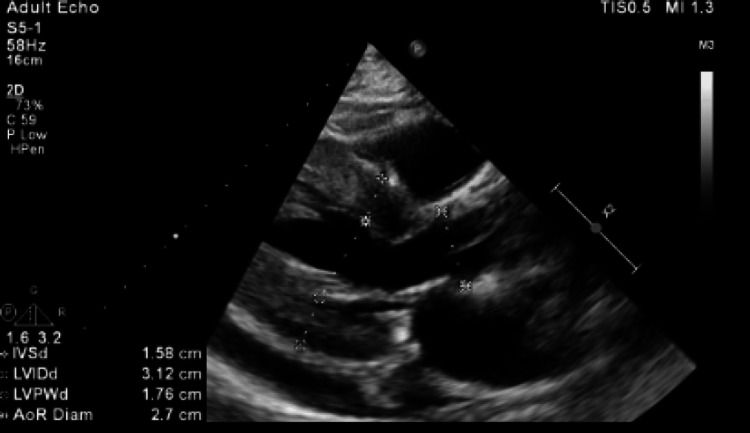
Patient's Echo Normal left ventricular dimension, severe concentric left ventricular hypertrophy

Echo was completed showing normal left ventricular dimension, severe concentric left ventricular hypertrophy, and normal systolic function (EF of 65-70), however, flow acceleration was noted at the left ventricular outflow tract (LVOT) and 40 mm outflow tract gradient. Analysis of mitral valve inflow, pulmonary vein Doppler, and tissue Doppler suggested grade 1a diastolic dysfunction with elevated left atrial pressure. The patient previously followed up with a cardiologist for HOCM and was already on metoprolol and verapamil, which could have led to decreased outflow obstruction on admission echo (Figure 2) (Videos 1, 2).

**Video 1:** Echo Normal left ventricular dimension, severe concentric left ventricular hypertrophy, and normal systolic function (EF of 65-70), however, flow acceleration was noted at the LVOT. Analysis of mitral valve inflow, pulmonary vein Doppler, and tissue Doppler suggested grade 1a diastolic dysfunction with elevated left atrial pressure.

**Video 2:** Echo 2 Sever Left Ventricular Hypertrophy

**Figure 3:**
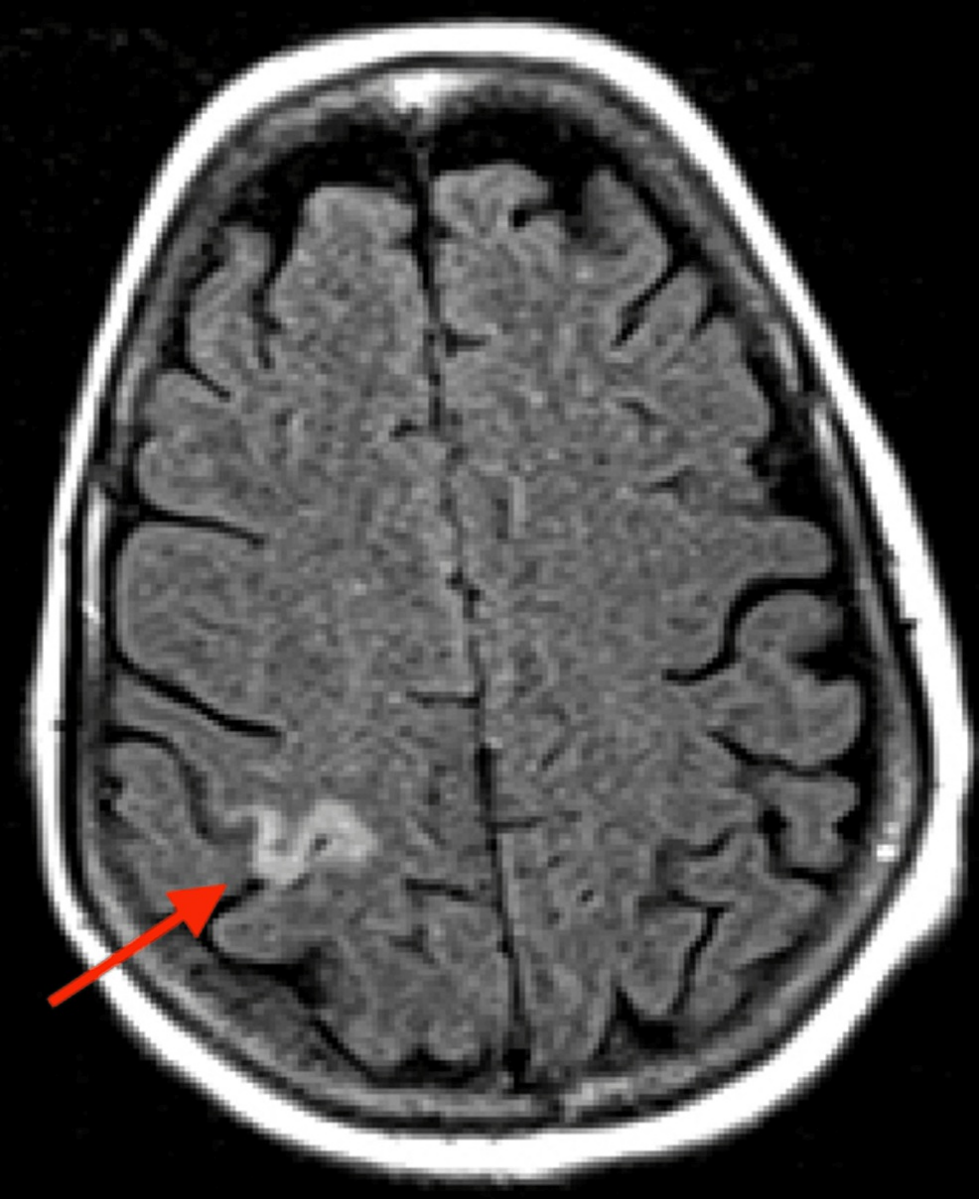
MRI axial flair, acute infarct involving high right parietal subcortical white matter.

**Figure 4:**
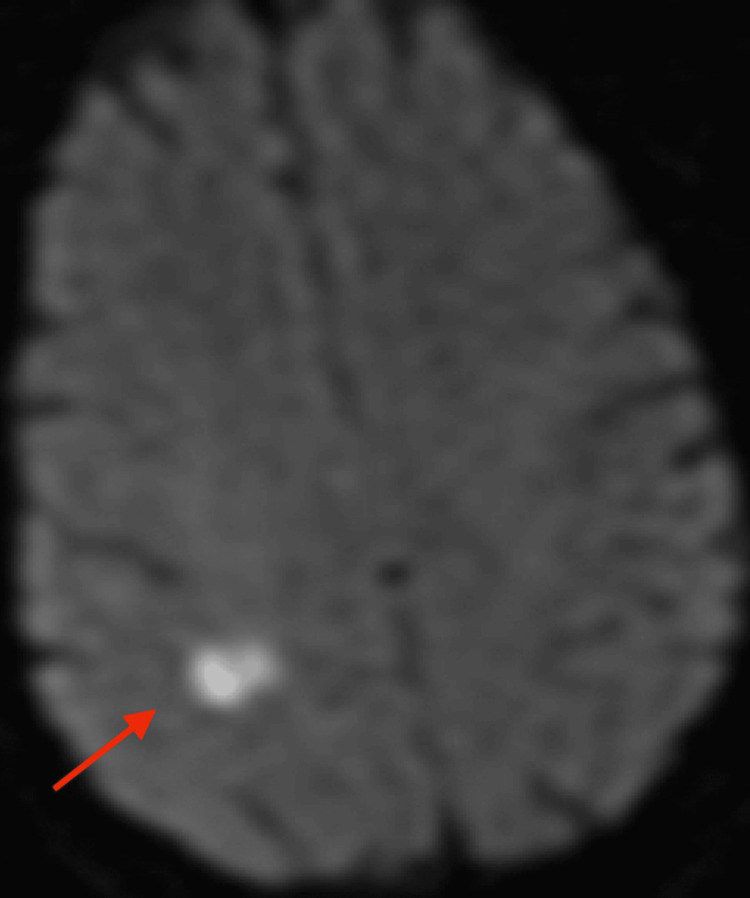
Brain MRI DWI axial Acute infarct involving high right parietal subcortical white matter. DWI: Diffusion-weighted imaging

**Figure 5:**
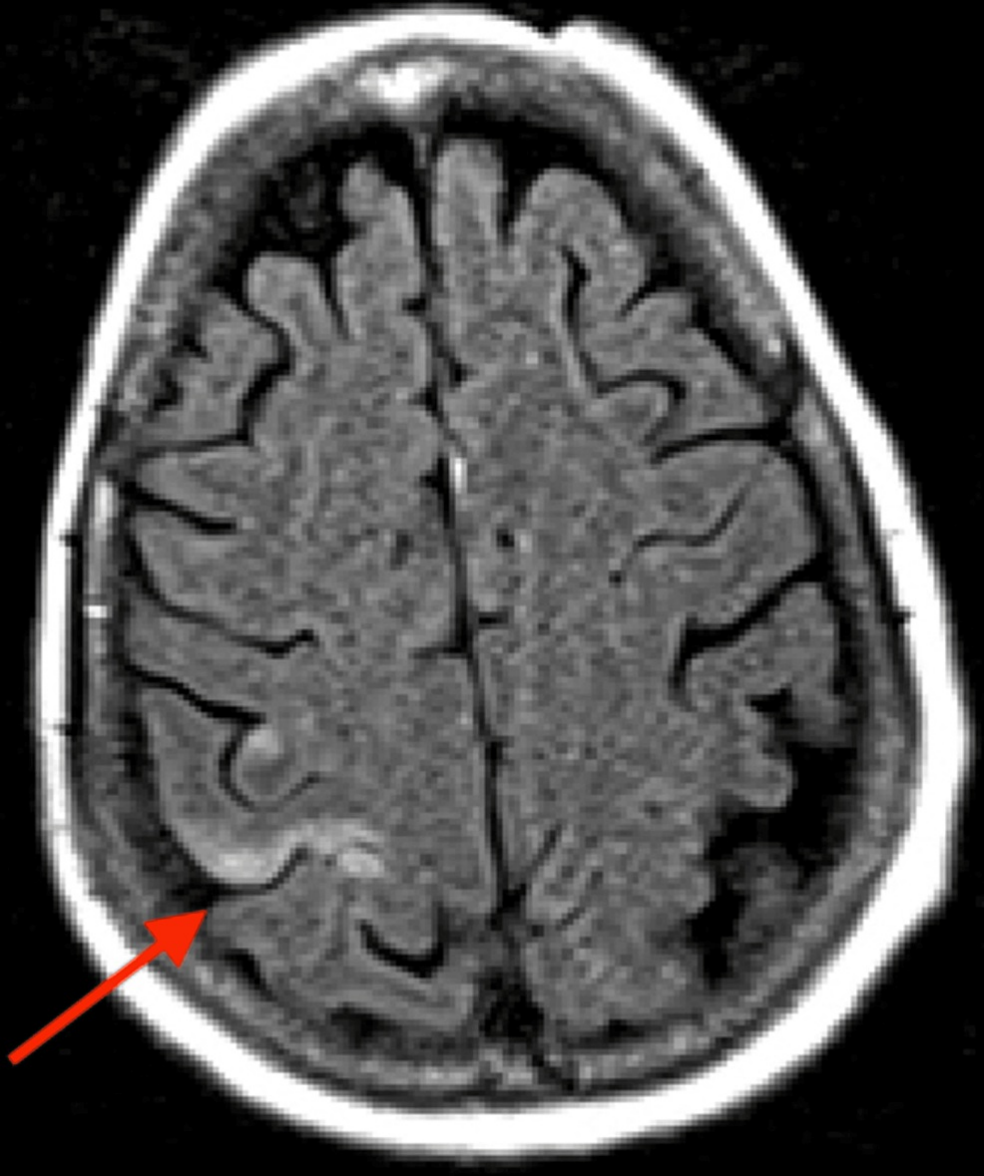
MRI axial flair, acute infarct involving high right parietal subcortical white matter

**Figure 6:**
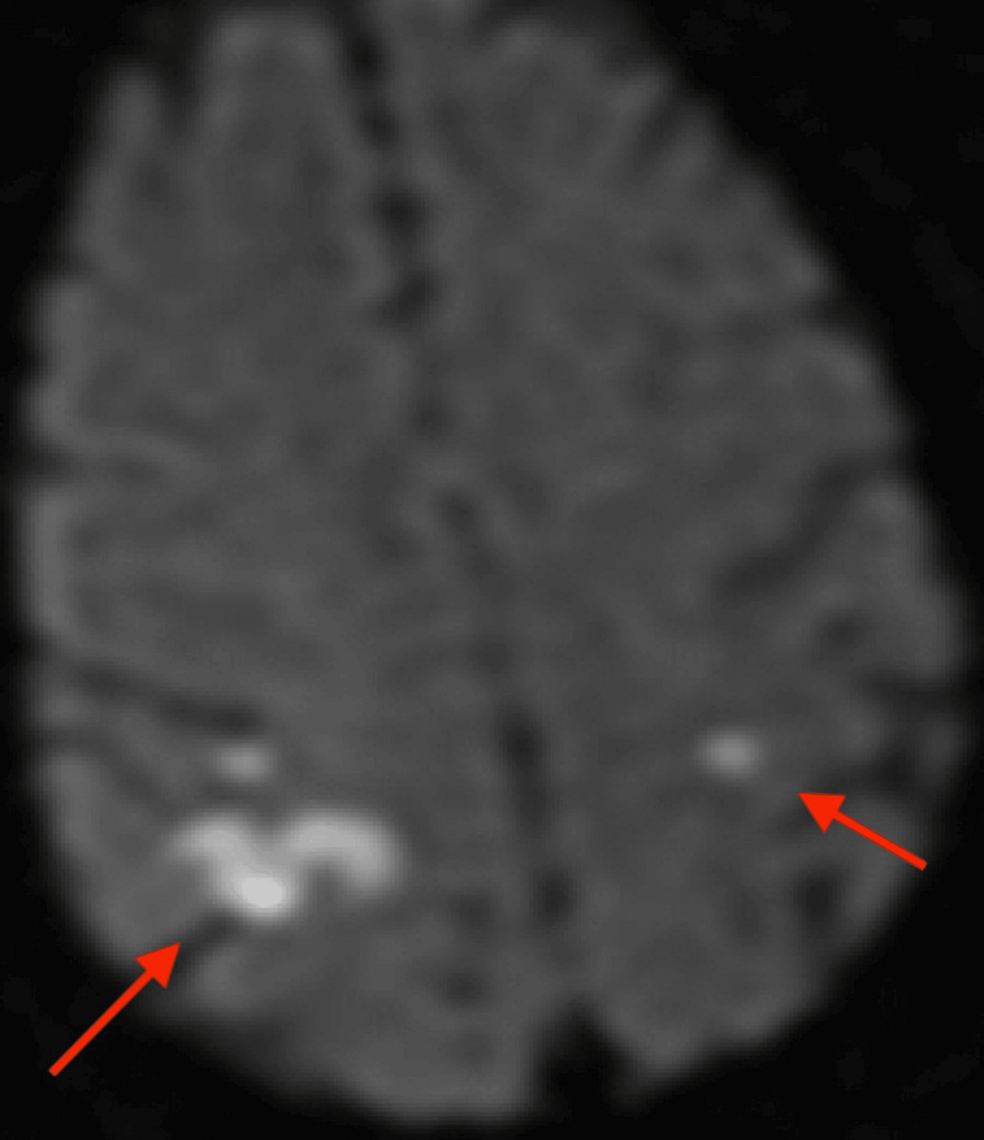
Brain MRI DWI axial Acute and subacute lacunar infarcts involving the high left posterior frontal and parietal subcortical white matter. DWI: Diffusion-weighted imaging

**Figure 7:**
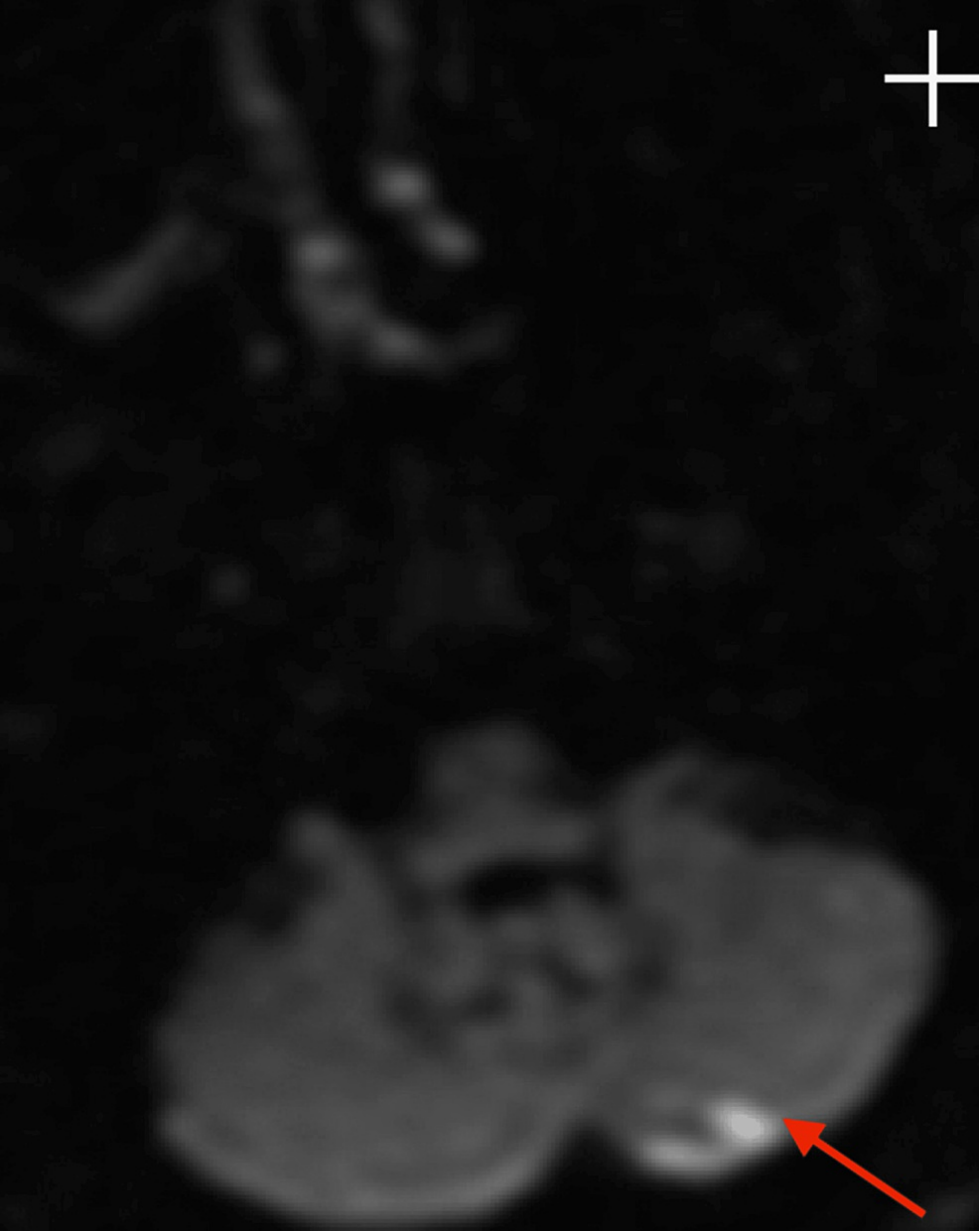
Brain MRI DWI Axial Acute infarct involving the medial left cerebellar hemisphere. DWI: Diffusion-weighted imaging

**Figure 8:**
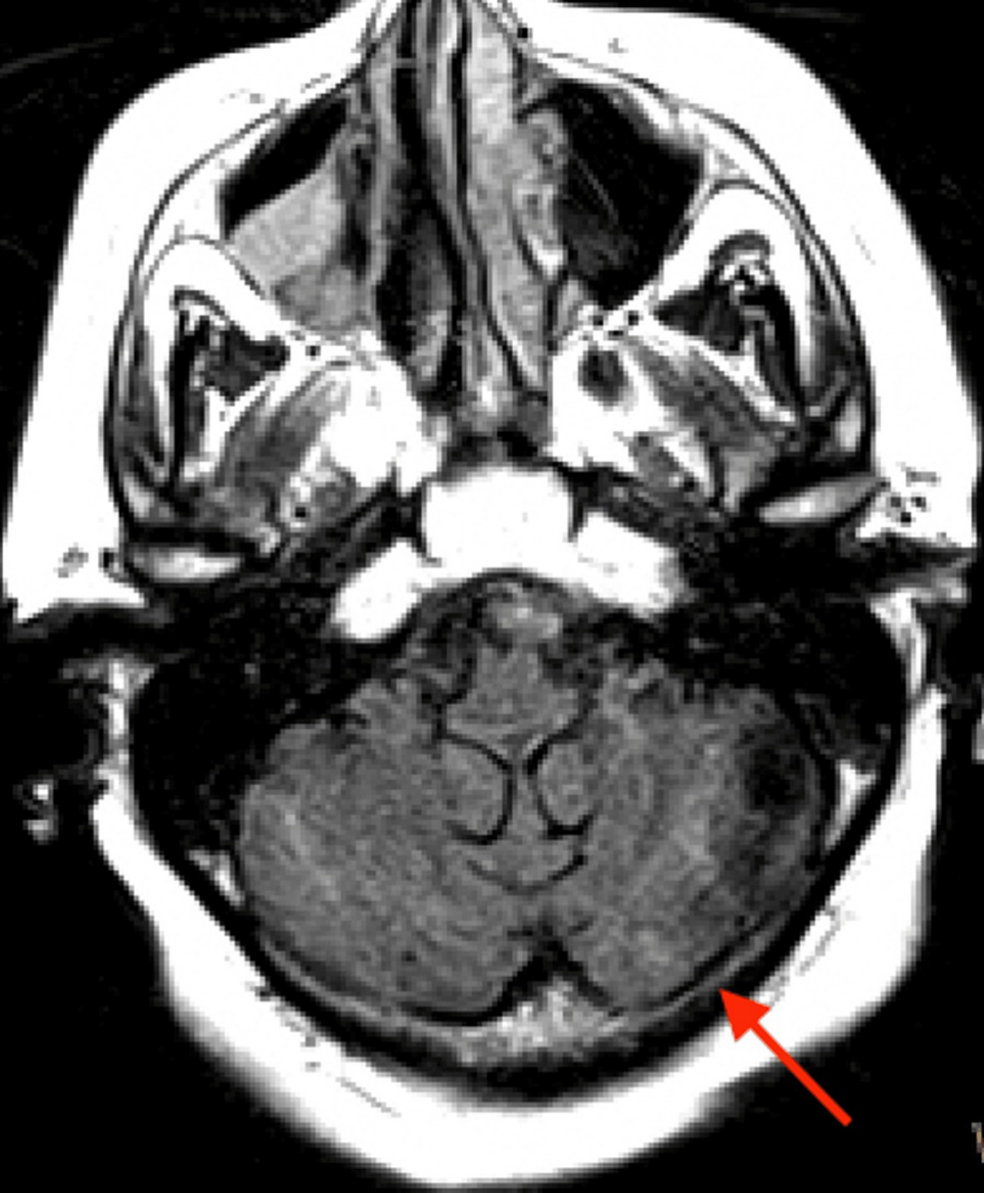
MRI axial flair, acute infarct involving the medial left cerebellar hemisphere.

However, brain MRI revealed small acute and subacute infarcts involving the high right parietal subcortical white matter and medial left cerebellar hemisphere. Acute and subacute lacunar infarcts involving the high left posterior frontal and parietal subcortical white matter were also present. MR findings noted to be bihemispheric, multiterritory infarcts in meeting with a cardioembolic source (Figures 3-8). Figure 6 illustrates bilateral infarcts, more acute at the right middle cerebral artery territory and subacute at the left middle cerebral artery territory.

Initial management and discharge without anticoagulation

Upon admission, the patient was managed for the cerebrovascular event and received Aspirin and Plavix, antiplatelet medications commonly used for stroke prevention [4]. However, the decision to initiate anticoagulation therapy was deferred due to the absence of a confirmed diagnosis of persistent atrial fibrillation (AFib). Typically achieved with oral anticoagulants like warfarin or direct oral anticoagulants, anticoagulation is recommended for stroke prevention in patients with persistent AFib [1]. In this case, the patient was discharged on Aspirin and Plavix and advised to follow up with Neurology and Cardiology for further evaluation, including using a loop recorder to assess for AFib.

Subsequent readmission and recurrent stroke symptoms

**Figure 9:**
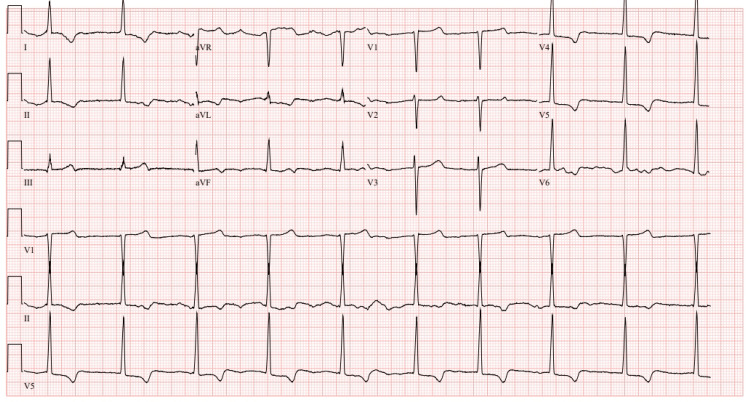
EKG on Discharge

The patient was readmitted shortly after the initial discharge due to hypertensive urgency and started on additional blood pressure medication to manage her high blood pressure [3]. However, the next day, she experienced transient palpitations, a pins and needles sensation in the right hand, and dysarthria, indicative of a possible transient ischemic attack (TIA). These symptoms prompted a stroke code, but they resolved by the time of evaluation. The patient had one paroxysmal episode of AFib on the telemetry monitor with no rapid ventricular response (RVR), which resolved spontaneously [4]. This finding reinforces the suspicion of underlying arrhythmias contributing to recurrent cerebrovascular events. Upon discharge, AFib had resolved and EKG on discharge was done, which showed Junctional rhythm, left ventricular hypertrophy, and VT of 57, no AFib noted (Figure 9).

Additionally, loop recorder results were not back yet, so the patient was again sent home without any anticoagulation, due to a lack of supporting evidence for anticoagulation for secondary stroke prevention in HOCM patients without a diagnosis of persistent AFib and a history of remote GI bleed. Differential diagnosis included infective endocarditis, LV thrombus, and autoimmune disease. Echo showed normal left ventricular dimension, severe concentric left ventricular hypertrophy, and normal systolic function (EF of 65-70), however, flow acceleration was noted at the LVOT. Analysis of mitral valve inflow, pulmonary vein Doppler, and tissue Doppler suggested grade 1a diastolic dysfunction with elevated left atrial pressure. The patient followed up with a cardiologist for HOCM and was already on metoprolol and verapamil, which could have led to decreased outflow obstruction on admission echo. Due to the patient's age (64), autoimmune, while on the differential for new onset of multiple strokes, seems less likely than embolic cause in the setting of paroxysmal AFib and HOCM.

Care was interrupted at that point as the patient switched care to another hospital, so no follow-up was obtained on whether or not the patient had any more recurrent CVA events or a diagnosis of persistent AFib was made.

## Discussion

Overview of studies and research on stroke risk in HOCM patients

Several studies have extensively investigated the risk of stroke in patients with hypertrophic obstructive cardiomyopathy (HOCM), particularly focusing on those with associated atrial fibrillation (AFib) [1]. The research consistently demonstrates a heightened risk of stroke in HOCM patients with persistent AFib. However, there remains a gap in specific studies focusing on the stroke risk in HOCM patients without confirmed persistent AFib, which necessitates further investigation.

Stroke and embolic events are known complications of hypertrophic cardiomyopathy (HCM) and are especially prevalent in the setting of atrial fibrillation, a common sequelae of this pathologic process. Due to the often paroxysmal and asymptomatic nature of atrial fibrillation, the relationship between atrial fibrillation and stroke in patients with hypertrophic cardiomyopathy is not well understood. However, there have been studies that attempt to analyze the risk of stroke in patients with hypertrophic cardiomyopathy without atrial fibrillation. A study conducted by Haruki et al. enrolled 593 patients with clinically diagnosed hypertrophic cardiomyopathy from 1980-2010 and investigated the prevalence of embolic events and evaluated risk factors in these patients without documented atrial fibrillation. Amongst the 431 patients without previously documented atrial fibrillation older age at diagnosis and left atrial dilation >48 mm were identified as independent determinants of embolic events. The incidence of stroke and embolic events was approximately 1.0% per year in the cohort and more than half of these patients had no previously documented history of atrial fibrillation [5]. A recognized limitation of this study is that it is possible that a subclinical or first paroxysmal episode of atrial fibrillation in patients without previously documented history may lead to a stroke or embolic event [5]. However, this limitation is addressed to some extent in a study conducted by Fumagalli et al., in which patients with hypertrophic cardiomyopathy with sinus rhythm confirmed by cardiac implantable electronic devices were analyzed. In hypertrophic cardiomyopathy patients with confirmed sinus rhythm, left atrial dilation >48 mm posed the greatest risk of stroke [6]. And in these patients with no prior history of atrial fibrillation, stroke rates were similar to those with de novo atrial fibrillation or stable sinus rhythm. Furthermore, cox multivariate analysis revealed that after adjustment for oral anticoagulation (OAC), left atrial dilation was independently associated with stroke while rhythm was not [6].

A large nationwide cohort conducted by Lin et al. observed the risk of ischemic stroke in patients with hypertrophic cardiomyopathy in the absence of atrial fibrillation with results comparable to those of matched general population with atrial fibrillation [7]. Hypertrophic cardiomyopathy patients without atrial fibrillation possess a high risk of stroke and anticoagulation therapy may be necessary. In the HCM cohort without AF, subjects had similar risk of ischemic stroke as the reference cohort, implying HCM might be a profound thromboembolic risk as AF [7]. Aging and other cardiovascular diseases cause atrial cardiomyopathy, which can result in AF and thromboembolism. The abnormal atrial substrate could promote thrombosis in the absence of AF. Once AF develops, the dysrhythmia causes contractile dysfunction and stasis, which further increases the risk of thromboembolism [8].

Studies on anticoagulation for stroke prevention in HOCM patients without confirmed persistent AFib

Although there is a dearth of studies directly addressing stroke prevention in HOCM patients without confirmed persistent AFib, some research has explored the potential benefits of anticoagulation in patients with cardiomyopathies, including HOCM, without persistent AFib [2]. SPAF1 study demonstrated that aspirin and warfarin were both effective agents in reducing ischemic stroke and systemic embolic events in patients with Afib when compared to placebo. Because warfarin-eligible patients composed a subset of all aspirin-eligible patients, the magnitude of reduction in events of warfarin vs aspirin could not be compared. Patients with nonrheumatic AFib who can safely take either aspirin or warfarin, where the benefit of stroke prevention outweighs the risk of bleeding, should receive prophylactic antithrombotic therapy to reduce the risk of stroke [9]. Additionally, per Risøe and Gjesdal, patients with paroxysmal atrial fibrillation have a risk of thromboembolic complications probably equivalent to those with permanent atrial fibrillation. Patients with a previous cerebral infarction, hypertension, age above 65, diabetes, previous myocardial infarction, reduced left ventricular function, heart failure or enlarged left atrium with or without a visible thrombus are especially prone to thromboembolic complications. International guidelines recommend anticoagulation therapy with warfarin to international normalized ratio (INR) levels between 2.0-3.0 for the majority of patients with atrial fibrillation [10]. Moreover, patients' symptoms are not a reliable surrogate parameter for the detection of AF. Moreover, antiarrhythmic therapy does not totally prevent atrial fibrillation, but raises the risk of silent AF episodes by reducing the mean heart rate. Based on these findings, effective anticoagulation should be taken into consideration in patients with paroxysmal AF independent of antiarrhythmic medication. The decision for anticoagulation with cumarine derivates or aspirin is dependent on the age, underlying diseases, and the individual thromboembolic risk in these patients [11]. Furthermore, per the ACTIVE study patients with paroxysmal AF treated with aspirin plus clopidogrel or OAC have a similar risk for thromboembolic events than patients with sustained AF. This risk can be significantly lowered with OAC [12]. As a counterargument, in patients with AF at moderate-to-high risk of stroke receiving anticoagulation, those with persistent AF have a higher risk of thrombo-embolic events and worse survival compared with paroxysmal AF [13]. While Choi et al. noted that HCM patients without documented AF are at a greater risk of ischemic stroke, especially in those 65 years of age or older or those with chronic heart failure [14].

These studies suggest that anticoagulation therapy is advantageous in reducing the risk of stroke in this patient population. However, further research is required to establish conclusive evidence and determine this subgroup's most appropriate anticoagulation strategies.

Current guidelines and recommendations

The current guidelines for stroke prevention in atrial fibrillation recommend using oral anticoagulants, such as warfarin or direct oral anticoagulants, for patients with persistent AFib [1]. However, these guidelines do not provide specific recommendations for stroke prevention in HOCM patients without confirmed persistent AFib. The absence of explicit guidelines underscores the need for further research and the development of evidence-based recommendations to manage stroke prevention in this patient population.

Discussion and arguments

Argument for Anticoagulation in HOCM Patients Without Confirmed Persistent AFib

Increased stroke risk: HOCM patients, even without confirmed persistent AFib, are still at an elevated risk of stroke due to underlying cardiac abnormalities and potential paroxysmal arrhythmias [1]. The hypertrophic cardiomyopathy (HOCM) condition itself can lead to blood flow abnormalities, promoting the formation of blood clots that may embolize the brain, causing a stroke [4]. Therefore, the risk of stroke is not solely dependent on the presence of confirmed persistent atrial fibrillation.

Shared mechanisms: The pathophysiology of stroke in HOCM patients without confirmed persistent AFib is similar to those with persistent AFib. The formation of thrombi in the atria can occur during paroxysmal arrhythmias, leading to embolic strokes [4]. These paroxysmal arrhythmias may go undetected without continuous monitoring. Thus, HOCM patients without persistent AFib may still be susceptible to the same thromboembolic complications as those with confirmed AFib [4].

Potential underdiagnosis of arrhythmias: It is possible that arrhythmias, including AFib, are underdiagnosed in HOCM patients without persistent AFib. Using loop recorders or prolonged monitoring may detect intermittent arrhythmias that could contribute to stroke risk [2]. Therefore, not relying solely on confirmed persistent AFib diagnosis may be crucial in identifying underlying arrhythmias and managing stroke risk effectively in HOCM patients.

Benefits of anticoagulation: Anticoagulation has proven efficacy in reducing stroke risk in patients with persistent AFib [1]. Extending the use of anticoagulation to HOCM patients without persistent AFib could offer similar benefits by reducing the formation of thrombi and subsequent embolic strokes. Considering the shared mechanisms and the potential underdiagnosis of arrhythmias [1,2], anticoagulation may be a reasonable intervention to mitigate stroke risk in this patient population.

Counterarguments Against Anticoagulation in HOCM Patients Without Confirmed Persistent AFib

Lack of evidence: The absence of specific studies addressing the use of anticoagulation in HOCM patients without confirmed persistent AFib makes it challenging to establish clear recommendations. With robust evidence, it is easier to determine the exact benefits and risks associated with anticoagulation in this specific patient population [3].

Bleeding risk: Anticoagulation carries a risk of bleeding, which may be a concern in patients without confirmed persistent AFib who have a lower stroke risk compared to those with persistent AFib. The potential bleeding complications associated with anticoagulant use should be carefully weighed against the anticipated benefits in stroke reduction, particularly in patients without confirmed persistent AFib [1].

Individualized approach: Some argue for an individualized approach, considering the patient's overall risk profile, including other risk factors for stroke, to determine the need for anticoagulation [1]. Assessing the patient's comprehensive risk factors, including age, comorbidities, and overall cardiovascular health, can help tailor the treatment plan and balance the potential benefits and risks of anticoagulation.

## Conclusions

In conclusion, the management of recurrent cerebrovascular events in patients with hypertrophic obstructive cardiomyopathy (HOCM) without confirmed persistent atrial fibrillation (AFib) poses a challenging clinical dilemma. Due to the often paroxysmal and asymptomatic nature of arrhythmias like AFib, they often go unrecognized. In addition to the underdiagnosis of arrhythmias, inherent increased stroke risk in HOCM patients may warrant anticoagulation therapy even in the absence of persistent atrial fibrillation. However, special consideration must be given to the lack of research on anticoagulation in this population as well as individual risk factors for bleeding. Given the complexity of this issue, further research is needed to provide definitive guidance on managing stroke prevention in HOCM patients without confirmed persistent AFib. It is crucial to conduct studies explicitly addressing this population to determine the most appropriate strategies for anticoagulation therapy and identify additional risk factors for stroke. Until then, a multidisciplinary approach involving cardiologists, neurologists, and other healthcare professionals is essential to individualize the management of stroke prevention in these patients based on their unique clinical profiles and shared decision-making.
